# HIV Delays IFN-α Production from Human Plasmacytoid Dendritic Cells and Is Associated with SYK Phosphorylation

**DOI:** 10.1371/journal.pone.0037052

**Published:** 2012-05-31

**Authors:** Calvin C. Lo, Jordan A. Schwartz, Dylan J. Johnson, Monica Yu, Nasra Aidarus, Shariq Mujib, Erika Benko, Martin Hyrcza, Colin Kovacs, Mario A. Ostrowski

**Affiliations:** 1 Department of Immunology, University of Toronto, Toronto, Ontario, Canada; 2 Department of Medicine, University of Toronto, Toronto, Ontario, Canada; 3 Maple Leaf Medical Clinic, Toronto, Ontario, Canada; 4 Li Ka Shing Institute, St. Michael’s Hospital, Toronto, Ontario, Canada; Blood Systems Research Institute, United States of America

## Abstract

Plasmacytoid dendritic cells (pDC) are the major producers of type I interferons (IFNs) in humans and rapidly produce IFN-α in response to virus exposure. Although HIV infection is associated with pDC activation, it is unclear why the innate immune response is unable to effectively control viral replication. We systematically compared the effect of HIV, Influenza, Sendai, and HSV-2 at similar target cell multiplicity of infection (M.O.I.) on human pDC function. We found that Influenza, Sendai, HSV-2 and imiquimod are able to rapidly induce IFN-α production within 4 hours to maximal levels, whereas HIV had a delayed induction that was maximal only after 24 hours. In addition, maximal IFN-α induction by HIV was at least 10 fold less than that of the other viruses in the panel. HIV also induced less TNF-α and MIP-1β but similar levels of IP-10 compared to other viruses, which was also mirrored by delayed upregulation of pDC activation markers CD83 and CD86. BDCA-2 has been identified as an inhibitory receptor on pDC, signaling through a pathway that involves SYK phosphorylation. We find that compared to Influenza, HIV induces the activation of the SYK pathway. Thus, HIV delays pDC IFN-α production and pDC activation via SYK phosphorylation, allowing establishment of viral populations.

## Introduction

The innate immune system is essential for the initial detection of invading viruses and subsequent activation of adaptive immunity. The type 1 interferons (IFNs) are the central effector cytokine of innate immunity and have been shown to inhibit HIV-1 (HIV) replication *in vitro* [1], [2], [3]. Plasmacytoid dendritic cells (pDC) are the major producers of type I IFNs in mice and humans. PDCs predominantly utilize TLRs for virus detection and IFN-α production [4], activation occurring through TLRs 7 and 9. Both of these receptors are endosomal and are triggered by ssRNA or double stranded CpG-DNA, respectively [5]. The IFN response by pDCs is both rapid and robust. With over 50% of the induced RNA transcripts following TLR7 and TLR9 triggering encoding for type I IFN, the result is production of 3–10 pg/cell of IFN-α protein at 24 h, 100-1000X more than any other cell type in the blood [6]. The enhanced capacity for pDCs to produce IFN is related to the high constitutive expression of IRF7 in pDCs, which allows for these cells to bypass the classic autocrine feedback involving IFN-beta (IFN-β) [7], [8].

Pathways downstream of TLR 7/9 signaling in pDCs bifurcates, leading to IFN-α production as well as maturation and inflammatory cytokine production [9], [10]. The pathway leading to IFN-α production depends on IRF7 phosphorylation and ubiquitination, following which IRF7 dimerizes, migrates to the nucleus, and facilitates transcription of IFN-α and IFN-β genes [9], [11], [12]. Maturation involves TNF Receptor Associated Factor (TRAF) 6 and the Interleukin-1 receptor-associated kinase (IRAK) 4, which mediates the downstream activation of NF-κB and MAPKs [10], [13]. NF-κB and MAPK activation result in pDC maturation, manifested by CD83, co-stimulatory molecule expression, and pro-inflammatory cytokine production, which include MIP-1α, IP-10, TNF-α, IL-6, IL-8, IL-10 and IL-12 [14], [15], [16], [17]. PDC maturation favors the priming of adaptive immunity resulting in T cell activation and inflammation [5], [6], [18]. Thus pDCs, through a dual signaling pathway, link innate immune responses to adaptive immune responses.

HIV has been shown to activate pDC directly, causing them to mature and produce IFN-α and TNF-α [18]. PDC activation via HIV has been shown to require interactions between CD4 on pDCs and HIV gp120, as well as internalization and interaction between HIV-RNA and TLR 7 [19]. In SIV infection, pDC are observed 1 day post-infection at the vaginal mucosa near sites of replicating virus, but are unable to control subsequent viral replication and dissemination [20]. These pDC were also shown to express the chemokines MIP-1α, MIP-1β, and MIP-3α, which could play a role in recruiting more pDC and activated CD4 T cells to be targeted for infection. It is currently unclear what role pDCs are playing during the early events following viral exposure.

Previous studies examining pDC responses to HIV *in vitro* have utilized high titers of virus, likely exceeding levels found *in vivo* [19], [21], [22]. As such, it is unclear whether pDC can actually undergo normal activation in response to doses of virus expected in the setting of sexual transmission. In addition, the kinetics of IFN-α production to HIV has not been previously studied, nor have there been detailed comparisons of pDC responses to other RNA viruses that do not cause chronic infections [19], [22]. HIV is a rapidly replicating RNA virus [23], with viral production rates of up to 5×10^4^ viruses/infected cell, a virus doubling time of 0.65 days and a mean basic reproductive ratio of 8.0 [24], [25]. These rapid replication kinetics could potentially overwhelm the inhibitory effects of type I IFNs if the pDC response is not adequately rapid or robust. In order to address these aspects, we compared the direct effect of HIV with a panel of common virus pathogens, many of which have similar replication kinetics to HIV [26].

## Results

### Influenza Virus Induces Greater IFN-α from pDC Compared to HIV

**Figure 1:**
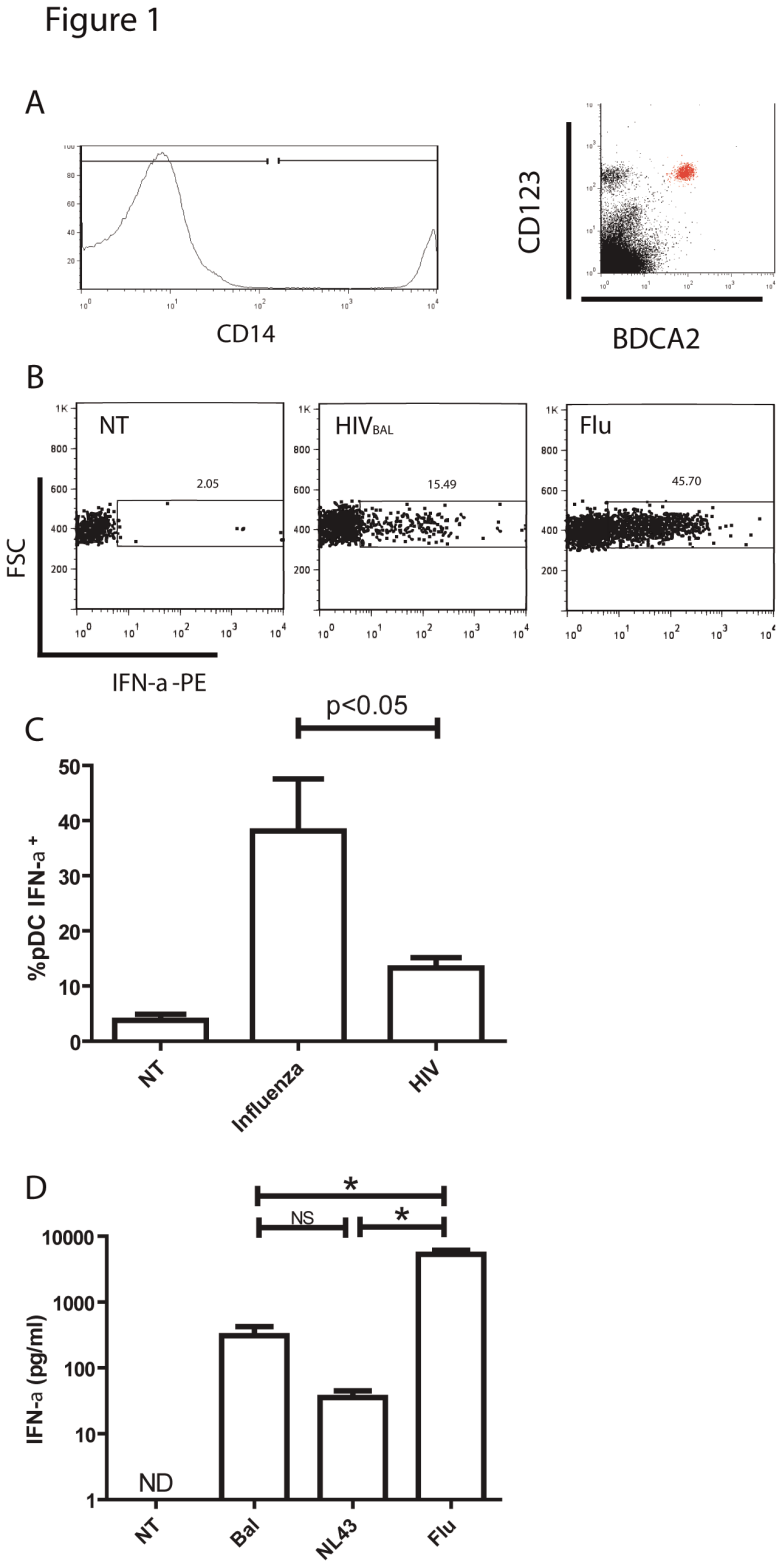
IFN-αproduction from pDCs following stimulation with HIV or Influenza. (A) Gating strategy used to identify CD14 negative BDCA-2 positive CD123 positive pDCs. (B) PBMCs from HIV negative individuals were treated with either Influenza (M.O.I = 0.1) or HIV_BAL_ (M.O.I = 0.1) in the presence of GolgiPlug™ for 8 hours and stained for intracellular IFN-α expression. Shown is a representative flow cytometry plot. (C) Summary data of pDCs from 3 individuals measuring intracellular IFN-α expression following stimulation with Influenza (M.O.I = 0.1) or HIV_BAL_ (M.O.I = 0.1) (D) Isolated pDCs from 3 HIV negative patients were stimulated with Influenza (M.O.I = 0.5) and HIV_BAL_ (M.O.I = 0.5) for 8 hours, and levels of IFN-α in supernatant were quantified by ELISA. Mean ± SEM, * = p<0.05.

We used a comparative approach to examine differences in pDC responsiveness to HIV and other viruses. In order to standardize for the effects of different viruses on human pDC, we used similar multiplicities of infection (M.O.I.s) of viruses, where the M.O.I. is calculated from the virus infections of their main target cell. In the first set of experiments, PBMC from healthy volunteers were co-cultured with HIV_BAL_, and compared to Influenza (both at an M.O.I. of 0.1) for 8 hours and then analyzed by flow cytometry for intracellular IFN-α production. PDCs were selected by positive staining for BDCA-2 and CD123, and negative staining for CD14 (Fig. 1A). A representative flow cytometry experiment is shown in Fig. 1B, with summary data from 3 individuals in Fig. 1C. We found that stimulation with influenza virus for 8 hours induced a greater percentage of IFN-α expressing pDC compared to HIV isolates at similar M.O.I.s. We then isolated pure populations of pDC by negative depletion of fresh PBMC from normal volunteers and co-cultured the pDC (purity 80–95%) with HIV_BAL_, HIV_NL-43_, or influenza [A/PR/8/34 (H1N1)] at an M.O.I. of 0.5 (for HIV, equivalent to 1.76 ng of p24 antigen) and measured IFN-α production in culture supernatant after 8 hours by ELISA. Summary data from 4 individuals, depicted in Fig. 1D further demonstrates that Influenza virus at comparable infectious doses is able to induce greater amounts of IFN-α in culture supernatants when compared to two strains of HIV.

### HIV Induces a Delayed IFN-α Response in pDC Compared to Other Viruses

**Figure 2:**
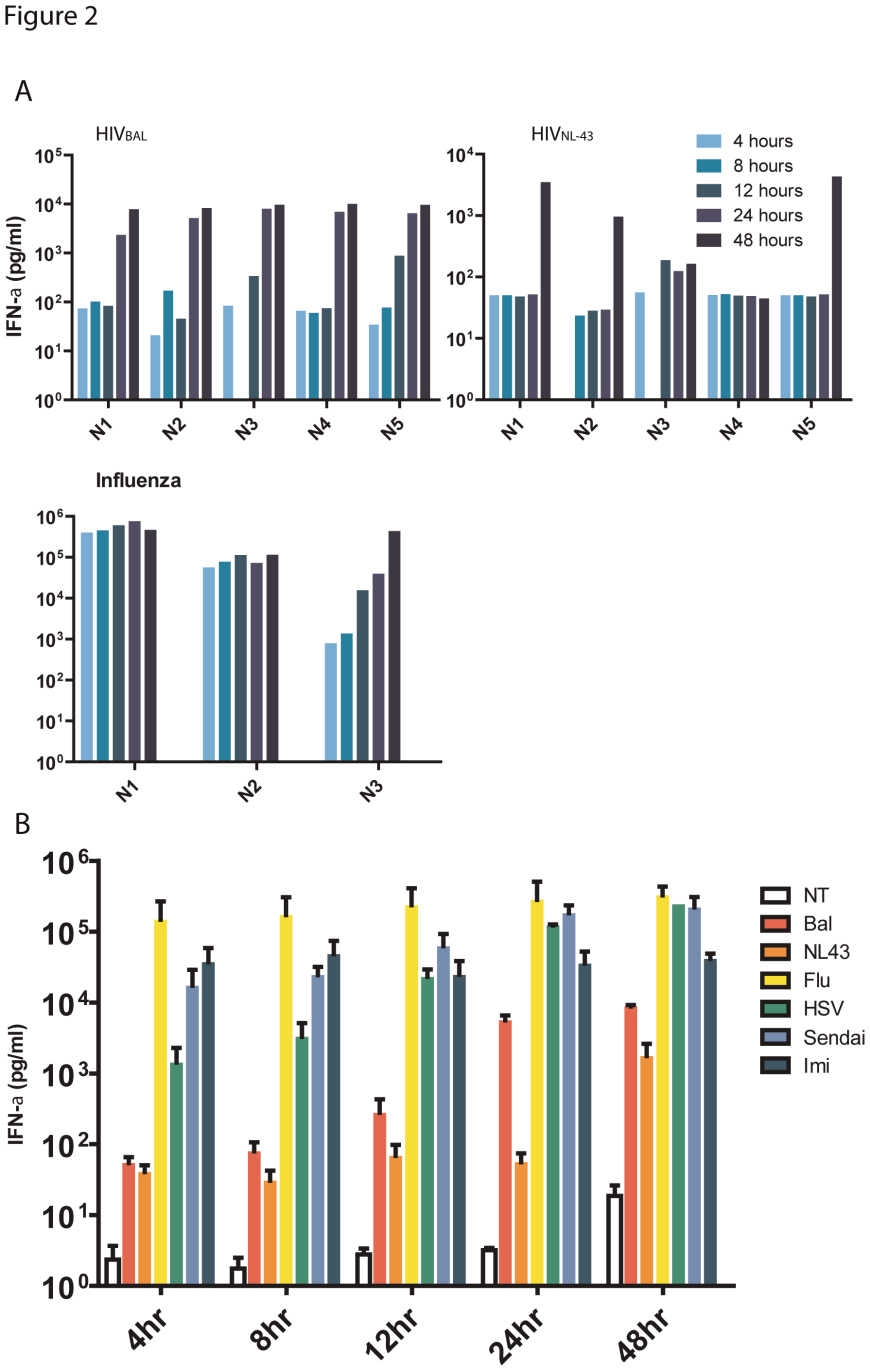
Kinetics of IFN-αproduction. Isolated pDCs from five HIV negative donors were rested overnight, and treated with a panel of stimuli as described in the materials and methods. Levels of IFN-α in supernatants were quantified by CBA as part of a panel of other cytokines. (**A**) Quantified IFN-α (pg/ml) present in culture supernatant of 3–5 donors (N1-N5) at various time points (represented by blue shaded bars) following stimulation by HIV_BAL_, HIV_NL43_ or Influenza (**B**) Mean ± SEM concentrations of IFN-α present in supernatants at various time points following stimulation by HIV_BAL_, (red), HIV_NL43_ (orange), Influenza (yellow), HSV-1 (green), Imiquimod (light blue), Sendai virus (dark blue), and medium control (white) from data from 5 donors.

**Figure 3:**
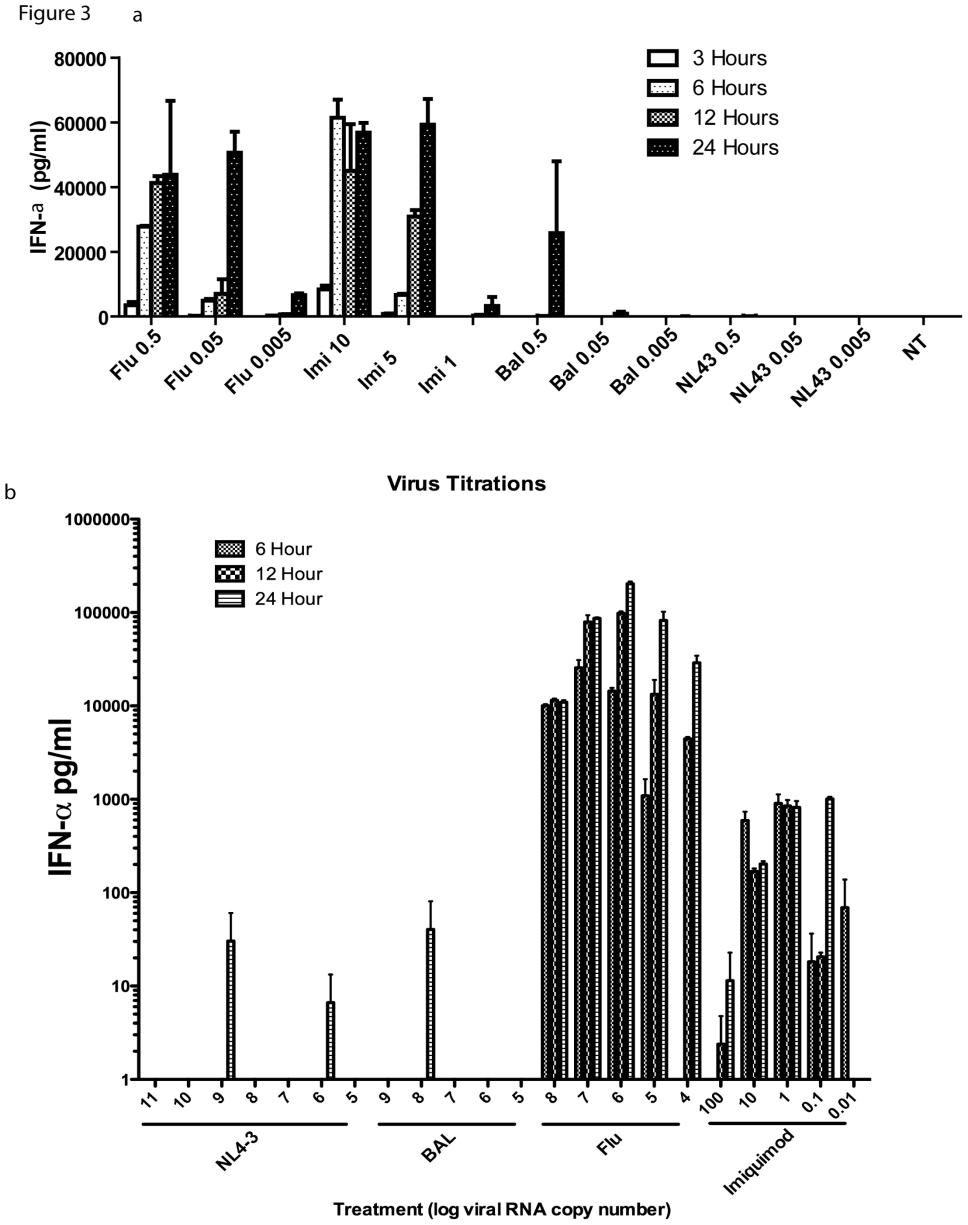
Response of pDC to varying amounts of viruses. 2×10^4^ isolated pDCs were plated in 96 well flat bottom plates overnight in R10+IL-3, then treated for designated time points with various concentrations of virus based on MOI or imiquimod (µg/ml) in a) and RNA copy numbers in b). Supernatants were then harvested and analyzed by ELISA for IFN-α. Each experiment is representative of one of two donors, performed in duplicate.

To compare the kinetics of IFN-α production from pDC after exposure to HIV and a larger panel of viruses, freshly isolated pDC from healthy volunteers were co-cultured with 0.5 M.O.I. of herpes simplex virus (HSV)-2, HIVBAL, HIVNL43, Influenza (H1N1), Sendai virus (18 HA units) and the TLR7 agonist imiquimod (20 µg/ml), and supernatants were sampled for IFN-α. Representative data from five individuals comparing HIV and Influenza are shown in Fig. 2A and summary data from all viruses from all individuals are shown in Fig. 2B. In addition, these differences were observed through a range of M.O.I.s and a range of RNA copy numbers of respective viruses (Fig. 3b and Table 1). These experiments demonstrate that RNA viruses such as Influenza, and Sendai induce IFN-α rapidly reaching about 90% of their maximal production by 4 hours of stimulation, whereas for HIV, maximum production of IFN-α was only achieved at 48 hours post virus exposure, at comparable RNA copy numbers. In addition, HIV viruses induced significantly less IFN-α even at maximal production when compared to Influenza, Sendai, HSV or imiquimod stimulation at similar target cell M.O.I.s.

**Table 1:** Viruses used for RNA quantitation.

	Infectious Particles	Protein (p24, or HA)	Theoretical Viral Particles	RNA copies	Nucleotides
HIV_BAL_	1.0×10^4^	1.76 ng p24	2.2×10^7^ *	2.47×10^8^	2.40×10^12^
HIV_NL43_	1.0×10^4^	2.92 ng p24	3.7×10^7^ *	3.99×10^10^	3.89×10^14^
Influenza A H1N1	1.0×10^4^	0.15 ng HA	3.6×10^6^ **	2.25×10^5^	3.06×10^9^

Standardized to an MOI of 0.5 on 20,000 pDCs.

* Based on 2000 p24 molecules/virus.

** Based on 400 HA molecules/virus.

### PDC Activation in Response to HIV is Delayed Compared to Other Viruses

**Figure 4:**
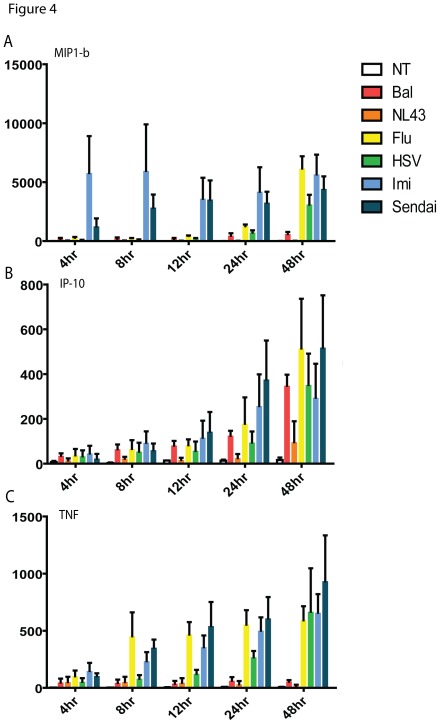
Cytokine expression profiles following stimulation. Isolated pDCs from HIV negative donors were rested overnight, and treated with a panel of stimuli as described in the materials and methods. Cytokines were quantified in culture supernatants by cytometric bead array. Mean ± SEM concentrations of (**A**) MIP-1β, (**B**) IP-10, and (**C**) TNF-α from 5 donors are shown at various time points following stimulation by HIV_BAL_, (red), HIV_ NL43_ (orange), Influenza (yellow), HSV (green), Imiquimod (light blue), Sendai virus (dark blue), and medium control (white). Neither IL-10 nor IL-12p70 was detected in any of the supernatant samples.

PDCs are capable of producing a range of cytokines and chemokines that may be involved in directing adaptive immune responses at the site of infection. As such, we used a cytometric bead array assay to examine the levels of MIP-1β, IP-10, TNF-α, IL-10 and IL-12 in pDC supernatants following stimulation with the panel of viruses. Examination of cytokine production in isolated pDC samples after virus exposure showed similarly delayed kinetics for both HIV isolates tested (Fig. 4). Influenza, Sendai, HSV and imiquimod induced MIP-1β, TNF-α and IP-10 with similar kinetics: MIP-1β appeared rapidly, particularly in imiquimod and Sendai stimulated samples (Fig. 4A), while TNF-α was detected at 12–24 hours post stimulation (Fig. 4C), and IP-10 was seen relatively later at 24–48 hours (Fig. 4B). In contrast, HIV tended not to induce comparable levels of cytokines over the time period measured, with the exception of IP-10. Neither IL-10 nor IL-12 was detected from pDC in any of the treatments. Thus, in comparison to other RNA viruses tested, HIV induced fewer pro-inflammatory cytokines.

**Figure 5:**
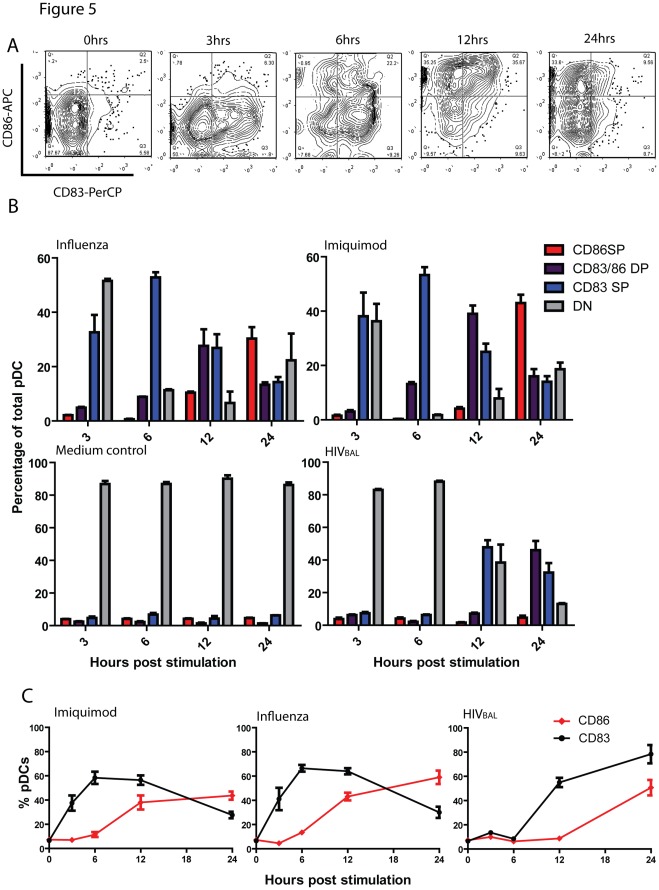
Kinetics of CD83 and CD86 upregulation. Isolated pDCs from HIV negative donors were treated with Influenza (M.O.I = 0.5), imiquimod (20 µg/ml), or HIV (M.O.I = 0.5) for various time points and harvested for analysis by flow cytometry for surface expression of CD83 and CD86. (A) Representative contour plots of CD83 and CD86 upregulation at various time points post stimulation by Influenza, showing distinct kinetics of CD83 and CD86 upregulation. (B) Percentage of pDCs that are CD83 single positive (SP, Blue bars), CD83/CD86 double positive (DP, Purple bars), CD86SP (Red bars), and double negative for both markers (DN, gray bars) at time points post stimulation with Influenza, imiquimod or HIV_BAL_. (C) Percentages of pDCs that are CD83^+^ or CD86^+^ at time points post stimulation. Data shown are representative of at least 3 different donors stimulated and stained in duplicate.

TLR signaling of pDC typically results in maturation of pDCs, antigen presentation, and expression of activation markers such as MHCII, CD83, CD80 and CD86 [19], [27], [28], [29], [30], [31]. As such, we tested whether HIV induced comparable levels of maturation in pDCs compared to Influenza or imiquimod. PDC were stained for activation markers, using similar co-culture conditions as described above. In Influenza stimulated samples, we observed early (at 3 hours) upregulation of CD83, followed by dual expression of CD83 and CD86, and subsequently downregulation of CD83 but persistent CD86 expression (24 hours) (Fig. 5). In HIV stimulated cells, there was markedly delayed expression of CD83 and CD83/86 co-expression (Fig. 5). Although an extended time course was not performed, it appears that HIV induced a similar sequence of CD83 followed by CD86 up-regulation, but with delayed kinetics. Thus, compared to imiquimod or other RNA viruses such as influenza, HIV delays pDC maturation.

### HIV Induces Elevated SYK Phosphorylation

**Figure 6:**
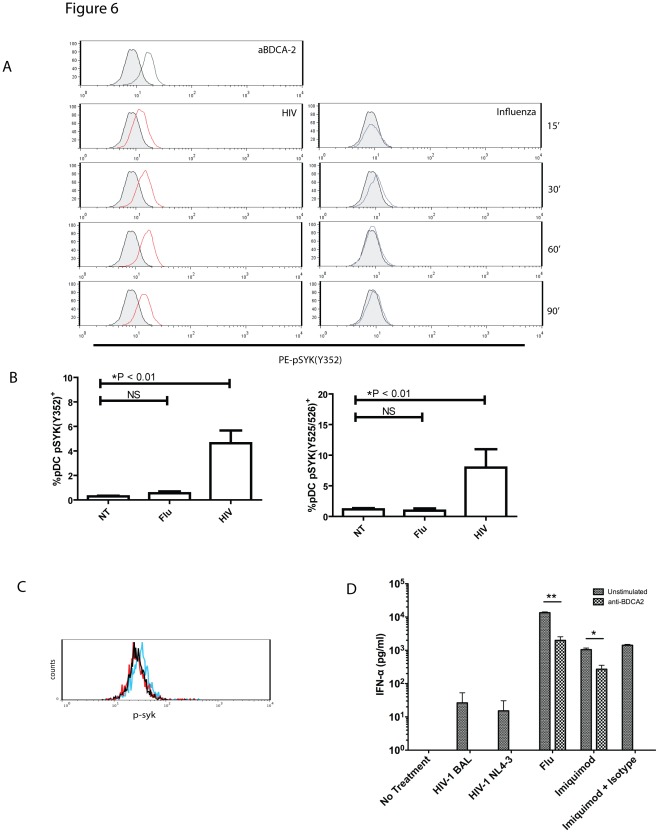
HIV induces upregulation of pSYK. (**A**) Purified pDCs were treated with Influenza and HIV_BAL_ for various time points. Positive control with anti-BDCA-2 antibody (1∶100) treated for 30 minutes is shown. Cells were harvested, permeabalized and stained for pSYK (Y352) as described in the materials and methods. Shown are representative histograms from 3 independent experiments. (**B**) PDCs were stimulated with HIV_BAL_, HIV_ NL43_, Influenza, or medium alone for 30 minutes and percentages of pDCs staining positive for pSYK (Y352) and pSYK (Y525/526) were evaluated by flow cytometry (n = 3). In (**C**) gp120 induces pSyk signaling in pDCs. Purified pDCs were stimulated for 90 minutes in the presence of 1 mg/ml monomeric gp120, AN1, (blue); 20 µg/ml imiquimod, (red); or the absence of stimulation (black). Cells were harvested and stained for pSyk (pY348) and analyzed by flow cytometry. Shown is a representative flow cytometric plot of two experiments. (**D**) pDCs were stimulated with 0.5 M.O.I. of HIV_BAL_, HIV_ NL43_, Influenza, and imiquimod, or medium alone for 24 hours in the presence or absence of anti-BDCA2 antibody or an IgG1 isotype control and IFN-α was measured by ELISA (n = 2, performed with two independent replicates, *p<0.001, **p<0.00005). BDCA2 treated pDC completely abrogated IFN-α production in the presence of HIV (bars not seen).

BDCA-2 has been identified as an inhibitory receptor on pDCs: ligation of BDCA-2 results in both diminished IFN-α responses as well as ineffective maturation [32], [33], [34] and HIV gp120 has previously been shown to ligate BDCA-2 [35]. Thus, we sought to determine whether the suboptimal IFN-α production, diminished cytokine production and delayed maturation could be a result of signaling through BDCA-2. BDCA-2 signaling utilizes a BCR-like signaling cascade involving SYK phosphorylation [36]. Using antibody specific to phosphorylated SYK (Y352), we demonstrate upregulation of intracellular phosphorylated SYK in pDCs within 15 minutes of exposure to HIV_BAL_ virus (Fig. 6A). This upregulation is sustained up to 90 minutes, at levels comparable to pDCs stimulated with cross-linking anti-BDCA-2 antibody. In contrast, Influenza stimulated cells did not demonstrate any appreciable increases in intracellular SYK levels. Previous reports indicate that SYK in pDCs is phosphorylated at multiple tyrosine sites, including Y352, Y525/526, and Y348. Using antibodies specific for phosphorylated SYK, we show that HIV induces phosphorylation of SYK at both tyrosine sites. In addition, monomeric gp120 treatment of pDC also induced SYK phosphorylation (Fig. 6C). Thus, HIV is capable of inducing SYK phosphorylation in pDCs, in part through gp120, indicating activation of the inhibitory BDCA-2 signaling cascade. To further demonstrate that BDCA2 signaling inhibits virus or TLR ligand induced IFN-α production, pDC were co-cultured with viruses or imiquimod in the presence of BDCA2 stimulating antibody or isotype control. All BDCA-2 stimulated conditions were associated with reduced IFN-α (Fig. 6D).

## Discussion

In this report, we provide evidence that HIV stimulation of pDCs induces sub-optimal responses compared to other viruses and stimuli in multiple areas, including cytokine profile, IFN-α production, and maturation kinetics. Furthermore, we provide data supporting a mechanistic explanation of these suboptimal responses, involving the inhibitory BDCA-2 signaling pathway.

The uniquely rapid and robust ability of pDCs to produce IFN–α implicates these cells as key players during early critical events of virus exposure. Although pDCs are rapidly recruited to the vaginal mucosa following intravaginal exposure to SIV, subsequent viral dissemination occurs [20], indicating potential defects in the pDC response to HIV/SIV. Although *in vitro* studies have repeatedly demonstrated the ability of HIV to induce IFN-α in pDCs, most of these studies have utilized high doses of HIV in the range of 300–900 ng/ml of p24 antigen [19], [21], [22]. These doses are likely unrepresentative of levels of virus that are sexually transmitted, as there is evidence that HIV infection is established primarily by a small founding population of virus [20], [37]. We attempted to use amounts of HIV in our experiments that estimated levels found during transmission *in vivo*. The amounts of virus used in our studies ranged from 10^8^ to 10^10^ RNA copies of HIV/experiment, which are similar in quantities used in sexual transmission studies of SIV infection of rhesus macaques [38]. Although it is difficult to accurately determine the amount of HIV required to directly transmit virus at the genital mucosa, the amounts used in our experiments were also within the range described in blood during acute HIV infection [39], and somewhat higher than observed in semen associated with a high rates of transmission [40], [41].

In order to provide a more reliable platform for comparison, we examined pDC responses to similar doses of different viruses. Through this method, we demonstrate that HIV is relatively suboptimal at IFN-α induction, with responses that are both delayed, and less robust. PDCs stimulated by Influenza produce IFN-α within 4 hours of exposure, emphasizing these cells as early mediators of immune responses. HIV has rapid replication kinetics with a half life of <6 hours; it is therefore conceivable that a 12 hour delay in the pDC response is sufficient to allow several rounds of replication to occur before IFN-α is able to exert its antiviral effects.

Similar defects in both maturation and cytokine production were observed when comparing HIV stimulated pDCs to pDCs stimulated by other viruses. Delays in the upregulation of CD83 and CD86 were seen, compared to Influenza, which is a robust activator of pDCs. CD83 and CD86 are typical markers used to gauge dendritic cell maturation as well as antigen presentation and T-cell activation capacity [15], [30], [42]. Despite this, the role of these markers in pDC mediated T-cell activation is still poorly defined. Remarkably, in both HIV and Influenza stimulated pDCs, the upregulation of CD83 and CD86 occurred with distinct kinetics; CD83 upregulation occurred early following stimulation and eventually declined, whereas CD86 upregulation occurred later following stimulation. The implications of these observations on recruitment and activation of T-cells at the sites of HIV exposure remains to be explored. In addition to maturation, we examined whether or not HIV induced comparable cytokine profiles compared to other stimuli. With the exception of IP-10, the levels of cytokine and chemokines induced by HIV were less robust compared to other viruses. Interestingly, IP-10 is involved in the recruitment of macrophages and T-cells [43] and has also been implicated in stimulating HIV viral replication [44].

Our finding that HIV tends to downregulate pDC production of IFN-α and cytokine in comparison to other viruses, questions the paradigm that immune activation by pDC is a driving force of viral replication in infected individuals [45]. Previous studies have used large doses of HIV, 20 to 100 fold more than used in our experiments to study interactions of HIV with pDC [46]. It is possible that the quantities of HIV used in our studies may more accurately reflect how pDC are interacting with HIV *in vivo*. Clearly, further work studying pDC activation *ex vivo* from recently infected individuals may help to delineate the role of pDC and immune activation during HIV infection.

Our results are consistent with recent studies by Gondois-Rey et al comparing pDC responsiveness to HCV, HIV, HSV and Influenza, in which viral treatments were normalized by genome copy number [47]. Remarkably, compared to Influenza or HSV, pDCs demonstrated diminished IFN-α responses to HIV as well as HCV, another virus that establishes chronic infection. Together with our results, these data suggests that pDC responses to HIV are in general less robust and less rapid compared to other viruses and stimuli. Recently, O’Brien et. al. demonstrated, that pDC pre-stimulated with HIV can continue to produce IFN-α and cytokines upon restimulation with HIV or other viruses and lose the capacity to become refractory compared to pre-stimulation with TLR agonists [48]. This is consistent with the notion that HIV may sub-optimally stimulate pDC upon initial exposure thus, making them less refractory to subsequent stimuli, a phenomenon that has been demonstrated with T cell stimulation [49].

Variations in pDC responsiveness can be accounted for by intrinsic differences in viral genomes. For instance, differences in the number of stimulatory sequences present in virus genomes may affect for the potency of pDC responses. Recently, specific uridine-rich sequences in the HIV genome have been shown to have TLR7 stimulatory capacity [50]; similar studies examining Influenza, HSV and other RNA viruses remain to be performed. Furthermore, the length of viral genome may dictate the amount of stimulatory material present. Influenza, for instance, has 8 single stranded RNA segments that in total are 13588 nucleotides in length [51]. HIV virions on the other hand each contain two copies of single stranded RNA that are around 9750 nucleotides in length each [52]. However, our experiments used 3 logs more HIV nucleotides than influenza raising the possibility that HIV is obstructing pDCs from mounting an effective response.

In addition to the above, we hypothesized that HIV may be involved in the active inhibition of the pDC response through ligation of BDCA-2. Ligation and cross-linking of the BDCA-2 receptor on pDCs inhibits both maturation and interferon production [36], [53] Furthermore, gp120 from HIV is capable of binding to BDCA-2 and abrogating pDC responsiveness [35]. BDCA-2 signaling utilizes a B-cell receptor signalosome, involving factors such as SYK [36], [53]. Upon measuring SYK phosphorylation, we demonstrate phosphorylation of SYK at multiple tyrosine sites following exposure to HIV and gp120, suggesting that HIV may actively hijack the BDCA-2 signaling pathway in order to subvert the normal rapid pDC response. Recently, hepatitis B surface antigen (HbsAg) has also been shown to ligate BDCA-2 [54], suggesting that inhibition of pDC responses may be an important factor in a variety of viral infections. However, the mechanisms downstream of BDCA-2 ligation that affect pDC signaling are still unknown. Further studies examining the phosphorylation of molecules involved in pDC activation in response to HIV will provide further insight into how HIV alters typical pDC responses.

## Materials and Methods

### Ethics Statement

Informed consent was obtained in accordance with the guidelines for conduction of clinical research at the University of Toronto and Maple Leaf Clinic institutional ethics boards. Written Informed Consent and study approval was provided by the institutional research ethics boards of the University of Toronto, Canada and of St. Michael’s Hospital, Toronto, Canada. Human samples were obtained through blood draw or leukopheresis of HIV negative donors.

### PBMC Stimulation and Surface Marker Expression

PBMCs were isolated by a Ficoll-Paque Plus gradient (Amersham Biosciences, Piscataway, NJ). PBMCs were plated at 1×10^6^ cells/well with 200ul of R10 media (RPMI 1640 medium with 10% FBS, L-glutamine, 100 units/ml Penicillin G and 100 µg/ml of Streptomycin (Gibco-Invitrogen, Carlsbad, CA) supplemented with 20 ng/ml of human recombinant IL-3 (R&D Systems, Minneapolis, MN) in 96-well flat-bottom plates.

Cells were stimulated with Influenza A/PR/8/34 (Charles River Laboratories, Wilmington, MA), or HIV_BAL_ for 8 hours at 37°C and 5% CO_2_. Subsequently, cells were harvested and pDCs were identified by staining for surface expression of BDCA-2 (BDCA-2-FITC, Miltenyi Biotec, Auburn, CA) and the absence of CD14 (CD14-APC, BD Pharmingen, San Diego, CA). Cells were also stained for the expression of CD83 (CD83-PerCP, Biolegend, San Diego, CA) and CD86 (CD86-APC, Biolegend,). Samples were analyzed on a FACSCalibur™ flow cytometer using CellQuest software (BD Biosciences, San Diego, CA) and analyzed by Flow Jo Software (Treestar, Ashland, Oregan). HIV viral stocks were generated and titred in HIV negative PBMCs as previously described [55].

### IFN-α ICS

PBMCs were stimulated as above, with the addition of GolgiPlug™ (BD Biosciences) at 7 hours prior to the end of incubation to block golgi transport. Following incubation, cells were harvested and stained for surface expression of BDCA-2 and CD123. Cells were then permeabalized with Cytofix/Cytoperm™ (BD Biosciences) and stained for intracellular IFN-α (IFN–α-PE, Chromaprobe, Maryland Heights, MO) in Perm/Wash Buffer™ (BD Biosciences) as per manufacturer’s instructions. Samples were analyzed by flow cytometry as described above.

### Isolation of pDCs

Purified pDCs were enriched by negative depletion using Miltenyi pDC Isolation Kit (Miltenyi Biotec) and LD columns (Miltenyi Biotec) following manufacturer’s instructions. Purity of the enriched cell population was evaluated by staining for surface expression of BDCA-2 (BDCA-2-FITC, Miltenyi Biotec) and CD123 (CD123-PE, Miltenyi Biotec). Purity ranged from 80–95%, and yields ranged from 0.06%-0.23%.

### IFN-α ELISA

Isolated pDCs were plated at 2–3×10^4^ cells/well with 100 ul of R10 (Gibco-Invitrogen) +20 ng/ml of IL-3 (R&D) in 96 well flat-bottom plates, and left overnight at 37°C, 5% CO2. PDCs were stimulated the next day with HIV_BAL_ (M.O.I = 0.5), HIV_NL43_ (M.O.I = 0.5), Influenza (M.O.I = 0.5) or media alone for 8 hours. Supernatants were harvested, transferred to V-bottom 96 well plates, centrifuged to remove cell debris, and stored at −20°C. IFN-α levels were quantified by a pan-specific IFN-α ELISA (Mabtech, Nacka Strand, Sweden) according to manufacturer’s instructions.

### HSV-2 Virus Generation

Vero cells (African green monkey kidney epithelial cells) generously provided by Scott Gray-Owen (University of Toronto, Toronto, ON) were cultured at 37°C in DMEM supplemented with 5% BS and grown on T25 flasks to 80% confluency. Cells were infected at an M.O.I of 0.1 with stock HSV-2, a gift from J. Newton (McMaster University, Hamilton ON) for 24–48 hours, or until detachment from plate surface was observed. Cells were harvested and sonicated with a probe sonicator, and virus was harvested from cell debris following centrifugation. Viral titer was determined by performing serial dilutions of virus stock on plated Vero target cells at 80% confluency in 6 well plates. Following 48 hours of infection, media was aspirated and cells were stained with 500 ul of crystal violet stain and plaques were counted.

### PDC Stimulation

The following viruses were used for pDC stimulation: Influenza A/PR/8/34 (H1N1, lot #4XAPR060202), Sendai Virus, Cantell strain (ATCC VR-907 Para-influenza 1, in amnioallantoic fluid) (lot #7Y050401B), both were obtained from Charles River Laboratories (Wilmington, MA). HIV isolates were obtained from the NIH AIDS Reagent Program. HSV-2 viruses were prepared as above. ID_50_s were obtained for the following viruses after culture in PHA stimulated PBMC blasts for HIV isolates, in chick egg cultures for influenza and sendai, and Vero cell cultures for HSV. Thus, MOI were determined based on their respective ID_50_s.

Purified pDCs were plated at 2×10^4^ cells per well in R10 (Gibco-Invitrogen) +20 ng/ml IL-3 (R&D) and rested at 37°C, 5% CO2 overnight. The next day, cells were stimulated with the following panel: a) 18 HA/ml Sendai Virus Cantell strain (Charles River), b) HSV-2 (M.O.I = 0.5), c) HIV_BAL_ (M.O.I = 0.5) d) Influenza (M.O.I = 0.5, Charles River), e) HIV_NL43_ (M.O.I = 0.5) and f) 20 mg/ml of imiquimod (Invivogen, San Diego, CA) [56]. Supernatants were harvested from individual wells into V-bottom 96 well plates at 4, 8, 12, 24, and 48 hours post stimulation, and spun down at 1500 rpm for 10 minutes to remove cell debris. Supernatants were stored at -20°C until analysis.

### Viral RNA Quantification

Quantification of HIV RNA copy numbers was determined using Cobas Ampliprep/Cobas Taqman HIV Test, version 2.0 (Roche Diagnostics, Pleasanton, CA). Determination of influenza RNA copies was performed using a TaqMan qPCR system against Flu Matrix 2 gene as previously described [57] and performed on an ABI Prism 7900HT Sequence detection System (Foster City, CA).

### Quantification of Cytokines by Cytometric Bead Array

Supernatants were thawed at room temperature, diluted appropriately, and the presence of a panel of cytokines was measured by BD Cytometric Bead Array Flex sets (BD Biosciences) according to the manufacturer’s instructions. Briefly, supernatants were diluted with Assay Diluent (BD Human Soluble Protein Master Buffer Kit), and incubated for one hour with a mixture of beads specific for detecting levels of IL-6 (BD, coordinate A7), IL-8 (BD, coordinate A9), IL-10 (BD, coordinate B7), IL-12p70 (BD, coordinate E5), TNF-α (BD, coordinate C4), IFN-α (BD, coordinate B8), MIP-1β (BD, coordinate B9) and IP-10 (BD, coordinate B5). Recombinant human protein standards of each cytokine were run along with each sample set. After initial incubation, a master mix containing PE-conjugated detection antibodies was added to the beads and the mixture was further incubated for 2 hours. The beads were then washed in Wash Buffer (BD Biosciences) and analyzed by flow cytometry. Standard curves were generated using FCAP Array software (Soft Flow, Burnsville, MN) and sample mean fluorescence was measured by Flow Jo Software (Treestar, Inc.).

### Phosflow Assay

Phosflow was performed to measure the phosphorylation state of intracellular SYK following stimulation. Purified pDCs were plated at 2×10^4^ cells per well in R10 (Gibco-Invitrogen) +20 ng/ml IL-3 (R&D), and stimulated with Influenza (M.O.I = 0.5, Charles River), HIV_BAL_ (M.O.I = 0.5), 20 mg/ml of imiquimod (Invivogen) or anti-BDCA-2 mAb (Miltenyi Biotec) for 15, 30, 60, and 90 minutes. Cells were then fixed in prewarmed BD Phosflow Fix Buffer I (BD Biosciences) and incubated at 37°C for 10 minutes then permeabalized with BD Phosflow Perm buffer III (BD Biosciences). Cells were then washed with PBS +2% FBS, and intracellular phosphorylated SYK was stained with the following antibodies: p-SYK (Y348) (p-SYK-PE, BD Biosciences), p-SYK (Y525/526 and Y352) (Cell Signaling Technologies, Beverly MA) followed by PE-anti-Rabbit IgG (Ebioscience, San Diego, CA).
